# An Anthocyanin-Enriched Extract from *Vaccinium uliginosum* Improves Signs of Skin Aging in UVB-Induced Photodamage

**DOI:** 10.3390/antiox9090844

**Published:** 2020-09-09

**Authors:** Kyungae Jo, Gi Yeon Bae, Kyoungwon Cho, Sung Sun Park, Hyung Joo Suh, Ki-Bae Hong

**Affiliations:** 1 Department of Integrated Biomedical and Life Science, Graduate School, Korea University, Seoul 02841, Korea; kyungae11@korea.ac.kr (K.J.); rldus530@naver.com (G.Y.B.); suh1960@korea.ac.kr (H.J.S.); 2 R&D Center, Chong Kun Dang Healthcare Corporation, Seoul 07249, Korea; ginseng333@ckdhc.com (K.C.); saintsun@ckdhc.com (S.S.P.)

**Keywords:** *Vaccinium uliginosum*, skin aging, UVB, winkles, mitogen-activated protein kinase (MAPK)

## Abstract

Accumulating evidence indicates that botanical extracts affect skin biophysical parameters, such as hydration, transepidermal water loss (TEWL), melanin index, erythema index, and wrinkle development. *Vaccinium uliginosum* extract contains a high level of anthocyanins as antioxidant and is ideal for use in dietary skin care products. Here, we assessed the photoprotective effects of dietary *V. uliginosum* extract in ultraviolet B (UVB)-irradiated hairless mice. Quantitative analysis of anthocyanin composition in the ethanol-extracted *V. uliginosum* sample was performed using high-performance liquid chromatography (HPLC). Skin parameter analysis and hematoxylin and eosin (H&E) staining were conducted on skin samples from UVB-irradiated hairless mice to evaluate the effects of *V. uliginosum* extract on skin conditions. In addition, skin mRNA and protein expression were assessed to characterize the molecular mechanisms underlying the effects of the anthocyanin-enriched extract on skin appearance and condition. Administration of the ethanol-extracted *V. uliginosum* sample caused significant changes in skin water-holding capacity, TEWL, wrinkle-related parameters, and epidermal thickness in UVB-irradiated hairless mice. In addition, oral administration of *V. uliginosum* attenuated the gene expression of matrix metalloproteinase (MMP) and increased levels of tissue inhibitor of metalloproteinase (TIMP) and antioxidant-related genes. Further, *V. uliginosum* administration downregulated inflammatory cytokine levels and UVB-induced phosphorylation of extracellular signaling regulated kinase (ERK), as well as Jun N-terminal kinase (JNK) and p38 protein levels. Oral administration of anthocyanin-enriched *V. uliginosum* extract can improve the appearance and condition of the skin following UV irradiation.

## 1. Introduction

The skin is an essential barrier organ that prevents dehydration and defends the organism against the external environment. Skin aging is caused by both intrinsic aging and external aging, such as age increases, hormonal changes, stress, as well as environmental pollution and sunlight [1,2]. Age-related skin changes such as fine wrinkles, atrophy, and transepidermal water loss can be considered as the cumulative effect of intrinsic factors, and skin with coarse wrinkles, loss of elasticity and rough-textured appearance is known to be caused by external environmental factors [3,4]. Photoaging by ultraviolet (UV) irradiation results in the degradation of the dermal collagen matrix by matrix metalloproteinase (MMP). Additionally, an increase in UV-induced reactive oxygen species (ROS) affects the phosphorylation of protein kinases in the mitogen-activated protein kinases (MAPK) signaling pathway [5]. MAPK signaling increases the activity of activator protein-1 (AP-1) and the expression of MMPs, which break down extracellular matrix (ECM) proteins, such as collagen, which provide tensile strength to the dermis [6]. Additionally, inflammatory factors are activated by ROS; this decreases the abundance of ECM proteins by increasing MMP or cyclooxygenase-2 while reducing the amount of the collagen precursor, procollagen [7].

Previous research on the photoaging process in skin has focused on identifying substances that prevent free radical damages and reduce collagen degradation. Anthocyanins, an antioxidant agent, are natural pigments classified into the group of flavonoids with antiaging and/or skin-protective properties and still continues to receive attention from consumers and food industries [8]. Anthocyanins with free radical-scavenging capacities are known to be associated with the levels of inflammation-related molecules including interleukins (ILs), nuclear factor-kappa B (NF-κB), and ECM components (collagen, hyaluronic acid, and elastin) in the skin tissues [9,10]. Hence, there is a lot of interest in anthocyanin-enriched extracts for the inhibition of skin aging.

*Vaccinium uliginosum* L. is a species in the genus *Vaccinium* (family Ericaceae). The known pharmacological effects of *V. uliginosum* include the regulation of blood vessels, dysentery, antigens, and diabetic retinopathy, while having potential anticancer effects. It has also been reported to have strong antioxidant properties as it contains a large amount of bioactive substances such as anthocyanins and flavanols [11,12]. However, to our knowledge, studies on the effects of *V. uliginosum* extract on ultraviolet B (UVB)-irradiated skin aging have not yet been performed. This study was designed to determine whether *V. uliginosum* extract enhances skin health via the regulation of the antioxidant enzyme system, MMPs and MAPK signaling. We used skin parameter analysis, histochemical staining, molecular techniques, and ELISA analysis to evaluate the effects of anthocyanin-enriched extract, in a dose-dependent manner.

## 2. Materials and Methods

### 2.1. Materials and Animals

The ethanol-extracted *V. uliginosum* samples used in the experiments were obtained from Chong Kun Dang Co., (Seoul, Korea). Hairless mice (SKH-1, 4-week-old male) were purchased from Orient Bio Inc. (Seungnam, Korea). The mice were housed in controlled rooms (21 ± 1 °C, 50% relative humidity) with light (12:12 h light:dark cycle). All experiments were approved by the Korea University Institutional Animal Care & Use Committee (KUIACUC-2019-0062). After adaptation to the housing conditions for 1 week, six mice were allocated to each group, with a total of seven groups.

### 2.2. Liquid Chromatography Analysis and Radical Scavenging Assay

The ethanol-extracted *V. uliginosum* sample was dissolved in 10% formic acid (Sigma-Aldrich Co., St. Louis, MO, USA) in distilled water, filtered through a 0.45 μm micropore membrane (Merck KGaA, Darmstadt, Germany), and injected into the HPLC equipment. Cyanidin-3-*O*-glucoside, delphinidin-3-*O*-glucoside, and malvidin-3-*O*-galactoside were detected by the Agilent HPLC system (Agilent, Waldbronn, Germany) with a diode array detector and separated on an analytical C18 Sunfire column (250 mm × 4.6 mm, 5 μm; Waters, Wexford, Ireland) at 30 °C with a flow rate of 1 mL/min. Eluent A was 10% aqueous formic acid (Sigma-Aldrich), and eluent B was 24.5% methanol (Thermo Scientific Fisher, Rockford, IL, USA), 24.5% acetonitrile (Thermo Scientific Fisher), 10% formic acid, and 50% distilled water. Monitoring was performed at 534 nm. The antioxidant activity of the ethanol-extracted sample was measured using 2,2′-azinobis (3-ethylbenzothiazoline-6-sulfonic acid) diammonium salt (ABTS, Sigma-Aldrich) and 2,2′-diphenylpicrylhydrazyl (DPPH, Sigma-Aldrich) assays. ABTS and DPPH radical-scavenging activities were measured using the method described by Brand-Williams and Re, respectively [13,14]. The radical scavenging activities of the extract are expressed as IC_50_, which is the concentration required to suppress the formation of radical by 50%.

### 2.3. Skin Photoaging and Treatment

Mice were orally administered ethanol-extracted *V. uliginosum* sample, 5 days per week. To induce photoaging, UV was irradiated to all groups, except the NOR group, three times per week. For UV exposure, a UV irradiator (BLX-254, Vilber Lourmat, Marne La Vallee, France) with UVB ultraviolet lamps (T-8M, Vilber Lourmat) was used. The dose used for the first week was 1 MED (minimal erythemal dose, 55–60 mJ/cm^2^), while the dose was increased to 2 MED for the second week, then 3 MED for the third week was 3 MED, and finally 4 MED from the fourth week to the end of the experiment. The amount of light was measured using a UV light meter (UV-340, Lutron, Taipei, Taiwan). The total UV dose during the experiment was 78 MED. In order not to be affected by immediate UV damage, UV irradiation was not performed 3 days prior to skin collection. The obtained skin tissue was used for downstream analysis.

### 2.4. Evaluation of Skin Water-Holding Capacity, Transepidermal Water Loss (TEWL), Erythema Value, and Skinfold Thickness

Skin water capacity was measured using the Corneometer^®^ CM825 (CK Electronic GmbH, Cologne, Germany). TEWL, which is closely related to the skin barrier effectiveness and moisturizing functions, was measured with the Tewameter^®^ (CK Electronic GmbH). Erythema values were measured using the Mexameter^®^ MX18 (CK Electronic GmbH), all of which were mounted on the Multi Probe Adapter^®^ MPA 5 (CK Electronic GmbH). Measurements of skinfold thickness were performed by pulling up the skin between the neck and hip along the central dorsal skin line and using a micrometer (293-821-30, Mitutoyo, Tokyo, Japan). The results of the measurements were presented by comparing the values before and after UV irradiation.

### 2.5. Evaluation of Skin Wrinkle Formation

After a sacrifice, the skin wrinkle formation on normal and UVB-irradiated mouse skin was collected using a Visoline^®^ Replica Full Kit (CK Electronic GmbH). Skin surface replicas were analyzed to determine wrinkle parameters. Total wrinkle area (mm^2^), number of wrinkles, mean wrinkle length (mm), wrinkle depth (mm), and mean wrinkle depth (mm) were measured with a skin wrinkle analyzer (Visoline^®^ VL 650, CK Electronic GmbH).

### 2.6. Histochemical Staining

After sacrifice, the back-skin tissue was removed from the mice and the slides with tissue sections embedded in paraffin were stained with hematoxylin and eosin (H&E) solutions (Sigma-Aldrich) according to the manufacture’s manual, and stained slides were observed and analyzed with a stereoscopic microscope at 63× magnification (Axio Zoom v.16; Carl Zeiss, Göttingen, Germany). For immunohistochemical (IHC) analysis, the tissue sections were incubated with antibodies against type I collagen (diluted 1:200; ab34710; Abcam, Cambridge, UK) overnight at 4 °C. Quantitative analysis of the immunoreactivities was performed using digital slide scanner and data analysis software (Motic China Group, Co., Ltd., Xiamen, China), following the manufacturer’s manual.

### 2.7. Real-Time Polymerase Chain Reaction (RT-PCR) Analysis

Total RNA samples were extracted from the skin via TRIzol^®^ reagent (Invitrogen, Carlsbad, CA, USA) DNase-treated RNA samples were reverse transcribed using SuperScript^®^ III reverse transcriptase (Invitrogen) with oligo d(T). Gene expression analysis was performed on the resulting complementary DNA (cDNA) using double-stranded DNA dye (SYBR^®^ green, Applied Biosystems, Foster City, CA, USA) with StepOne plus real-time PCR system (Applied Biosystems). The results were normalized to a reference gene, glyceraldehyde-3-phosphate dehydrogenase (GAPDH), using the CT (2^−∆∆CT^) method [15]. The primer sequences used for RT-PCR are as follows: *GAPDH* (NM_ 001289726.1), forward primer (FP) 5′-CAT CAC TGC CAC CCA GAA GAC TG-3′, reverse primer (RP) 5′-ATG CCA GTG AGC TTC CCG TTC AG-3′; *MMP2* (NM_008610.3) (FP) 5′-CAA GGA TGG ACT CCT GGC ACA T-3′, (RP) 5′-TAC TCG CCA TCA GCG TTC CCA T-3′; *MMP3* (NM_010809.2) (FP) 5′-TTC TGG GCT ATA CGA GGG CA-3′, (RP) 5′-CTT CTT CAC GGT TGC AGG GA-3′; *MMP9* (NM_013599.4) (FP) 5′-GCT GAC TAC GAT AAG GAC GGC A-3′, (RP) 5 ′-TAG TGG TGC AGG CAG AGT AGG A-3′, *TIMP1* (NM_001044384.1) (FP) 5′-TCT TGG TTC CCT GGC GTA CTC T-3′, (RP) 5′-GTG AGT GTC ACT CTC CAG TTT GC-3′, *TIMP2* (NM_011594.3) (FP) 5′-AGC CAA AGC AGT GAG CGA GAA G-3′, (RP) 5′-GCC GTG TAG ATA AAC TCG ATG TC-3′, collagen type I alpha 1 chain, *COL1a1* (NM_007742.4) (FP) 5′-CCT CAG GGT ATT GCT GGA CAA C-3′, (RP) 5′-CAG AAG GAC CTT GTT TGC CAG G-3′), hyaluronidase, *HYAL* (NM_008317.6) (FP) 5′-AAG TAC CAA GGA ATC ATG CC-3′, (RP) 5′-CTC AGG ATA ACT TGG ATG GC-3′, superoxide dismutase, *SOD1* (NM_011434.2) (FP) 5′-GGT GAA CCA GTT GTG TTG TCA GG-3′, (RP) 5′-ATG AGG TCC TGC ACT GGT ACA G-3′), catalase, *CAT* (NM_009804.2) (FP) 5′-CAT TCG ATC TCA CCA AGG TTT G-3′, (RP) 5′-GGT AGG GAC AGT TCA CAG G-3′), glutathione peroxidase, *GPx* (NM_008160.6) (FP) 5′-CGC TCT TTA CCT TCC TGC GGA A-3′, (RP) 5′-AGT TCC AGG CAA TGT CGT TGC G-3′.

### 2.8. Western Blot Analysis

To evaluate the effects of anthocyanin-enriched extract on the levels of extracellular signal regulated kinase (ERK), c-Jun *N*-terminal kinase (JNK), and p38 phosphorylation, mouse skin tissues were homogenized and protein concentration was quantified using the bicinchoninic acid (BCA) assay kit (Thermo Scientific Fisher). Further, 25 μg of lysate was denatured with precast Tris-glycine gel (Bio-Rad, Hercules, CA, USA). Protein levels were detected with a specific antibody using the ChemiDoc TM imaging systems (Bio-Rad), results were normalized to the control protein, GAPDH, and primary antibodies were purchased from Cell Signaling Technology, Inc. (Danvers, MA, USA).

### 2.9. Cytokine Assay

For the cytokine assay, skin tissue fragments immersed in PBS containing the protease inhibitor cocktail (Sigma-Aldrich) were homogenized using a TissueLyser II (Qiagen, Venlo, The Netherlands). Protein concentration of the supernatant was quantified using a BCA assay kit (Thermo Scientific Fisher). Inflammatory cytokine assay kit (BD Bioscience, San Jose, CA, USA) was used to analyze cytokine levels of UVB-irradiated mouse skin, according to the manufacturer’s instructions.

### 2.10. Statistical Analysis

Data were expressed as mean ± standard deviation (SD) or standard error of the mean (SEM), and differences between groups were assessed through one-way analysis of variance (ANOVA) and Tukey’s multiple comparison test at a significance level of 0.05 by Statistical Package for Social Sciences version 25.0 (SPSS Inc., Chicago, IL, USA).

## 3. Results

### 3.1. Anthocyanin Content and Antioxidant Activity of V. uliginosum Extract

The anthocyanin content and antioxidant activity of the ethanol-extracted *V. uliginosum* sample are shown in Table 1. The polyphenols content of ethanol-extracted *V. uliginosum* sample was 44.37 ± 0.30 μg/mg and the cyanide-3-*O*-glucoside content was 127.9 ± 16.10 μg/g after the ethanol extraction procedure. After ethanol extraction, the content of delphinidin-3-*O*-glucoside and malvidin-3-*O*-galactoside were 572.14 ± 73.03 and 53.62 ± 7.81 μg/g, respectively. In addition, after ethanol extraction, the antioxidant activities were measured using ABTS and DPPH radical-scavenging assays and the ferric reducing/antioxidant power (FRAP) assay. ABTS and DPPH radical-scavenging abilities of ethanol-extracted *V. uliginosum* sample were 1.93 ± 0.11 and 2.44 ± 0.09 mg/mL, respectively. In addition, FRAP activity was 0.20 ± 0.00 mM. The loss of anthocyanins, which is susceptible to heat damage, was minimized by the extraction process. Thus, the ethanol-extracted *V. uliginosum* sample showed excellent antioxidant activity.

### 3.2. Effects of an Anthocyanin-Enriched Extract from V. uliginosum on Skin Barrier Function

**Figure 1:**
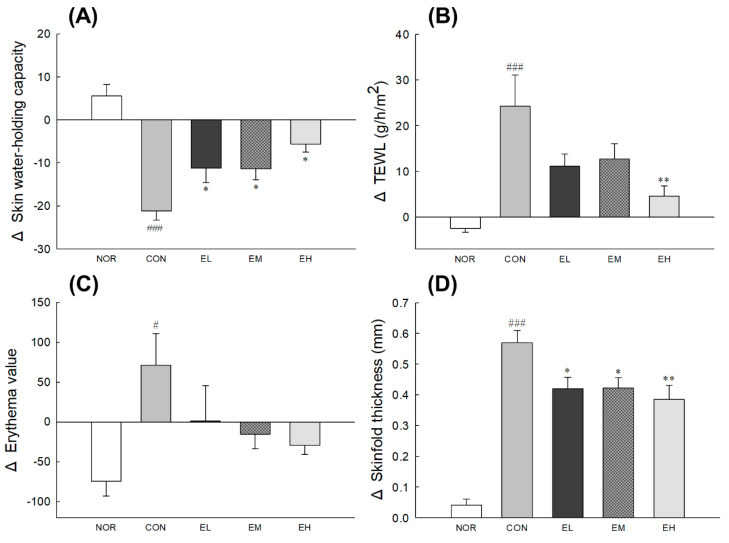
Effects of an anthocyanin-enriched extract from *Vaccinium uliginosum* on (**A**) skin water-holding capacity, (**B**) transepidermal water loss (TEWL), (**C**) erythema value, and (**D**) skinfold thickness in ultraviolet B (UVB)-irradiated mouse skin. NOR: normal group; CON: UVB-control group; EL: low dose of ethanol-extracted *V. uliginosum-*treated group; EM: middle dose of ethanol-extracted *V. uliginosum-*treated group; EH: high dose of ethanol-extracted *V. uliginosum-*treated group. Values are means ± standard error of the mean (SEM) for each group. Symbols indicate significant differences at ^#^ *p* < 0.05 and ^###^ *p* < 0.001 vs. NOR group and * *p* < 0.05 and ** *p* < 0.01 vs. CON group.

To evaluate the effect on skin water-holding capacity, TEWL, erythema value, and skinfold thickness of *V. uliginosum*, the dorsal skin of mice from each test group was assessed. In addition, the food intake, drinking volume, and average weight gain of mice were recorded twice a week during the experimental periods, and all the groups did not show any significant difference (Table S1). The skin water-holding capacity, TEWL, erythema value, and skinfold thickness were significantly changed in the UVB-control group (CON) compared to the NOR group, which did not receive UV radiation (Figure 1A,B,D: *p* < 0.001; Figure 1C: *p* < 0.05). Compared to the CON group, treatment groups showed significant difference in skin water-holding capacity (Figure 1A: *p* < 0.05), and the EH group had significantly altered TEWL (Figure 1B: *p* < 0.01). There was no significant difference in erythema value in the *V. uliginosum*-treated groups (EL, EM, EH) compared to CON group. As shown in Figure 1D, administration of an anthocyanin-enriched extract from *V. uliginosum* significantly decreased the skinfold thickness (EL and EM: *p* < 0.05; EH: *p* < 0.01).

### 3.3. Effects of an Anthocyanin-Enriched Extract from V. uliginosum on UVB-Induced Wrinkle Formation

**Figure 2:**
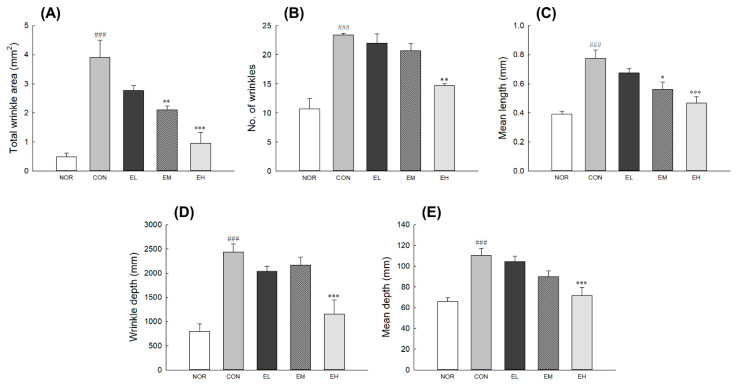
Effects of an anthocyanin-enriched extract from *Vaccinium uliginosum* on (**A**) total wrinkle area, (**B**) number of wrinkles, (**C**) mean wrinkle length, (**D**) wrinkle depth, and (**E**) mean wrinkle depth in UVB-irradiated mouse skin. NOR: normal group; CON: UVB-control group; EL: low dose of ethanol-extracted *V. uliginosum-*treated group; EM: middle dose of ethanol-extracted *V. uliginosum-*treated group; EH: high dose of ethanol-extracted *V. uliginosum-*treated group. Values are means ± standard error of the mean (SEM) for each group. Symbols indicate significant differences at ^###^ *p* < 0.001 vs. NOR group and * *p* < 0.05, ** *p* < 0.01, and *** *p* < 0.001 vs. CON group.

To assess the effects of *V. uliginosum* on skin wrinkle formation, replicas were taken from the central dorsal skin of mouse (Figure S1). After exposure to UVB irradiation, the dorsal skin showed erythema and thick wrinkles and appeared to cause slight damage on the surface. Skin parameters such as wrinkle area, number, depth, and length of CON increased significantly than NOR (Figure 2: *p* < 0.001). Treatment with *V. uliginosum* in the EH group significantly lowered all indicators of wrinkle formation compared to the CON group (Figure 2A,C–E: *p* < 0.001; Figure 2B: *p* < 0.01). In the EM group, total winkle area and the mean length of the skin surface were significantly less than in the control group (Figure 2A: *p* < 0.01; Figure 2C: *p* < 0.05). There was no significant difference in the EL group when compared to the CON group.

### 3.4. Effects of an Anthocyanin-Enriched Extract from V. uliginosum on Epidermal Thickness and Collagen Fibers

**Figure 3:**
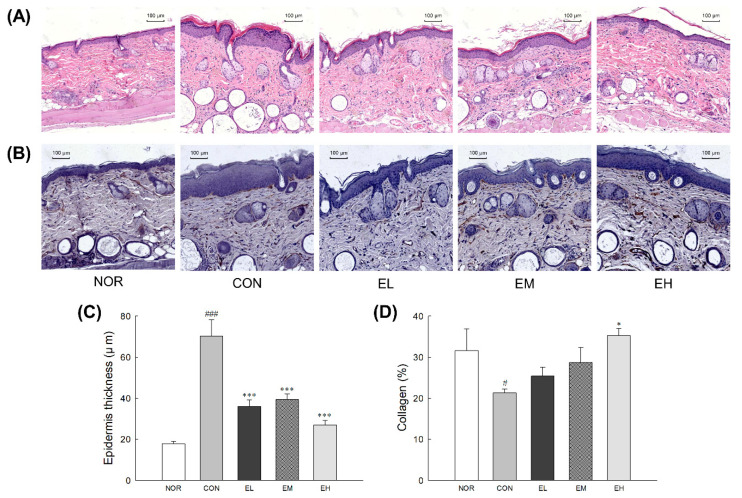
Effects of an anthocyanin-enriched extract from *Vaccinium uliginosum* on histological alteration of epidermal thickness (**A**,**C**) and collagen degradation (**B**,**D**) in UVB-irradiated mouse skin. NOR: normal group; CON: UVB-control group; EL: low dose of ethanol-extracted *V. uliginosum-*treated group; EM: middle dose of ethanol-extracted *V. uliginosum-*treated group; EH: high dose of ethanol-extracted *V. uliginosum-*treated group. Values are means ± standard error of the mean (SEM) for each group. Symbols indicate significant differences at ^#^ *p* < 0.05 and ^###^ *p* < 0.001 vs. NOR group and * *p* < 0.05 and *** *p* < 0.001 vs. CON group.

To evaluate epidermal thickness and collagen fibers, histological changes and collagen immunoactivity were visualized by H&E and IHC staining (Figure 3). UVB irradiation significantly increased the skin thickness of the CON group compared to the NOR group (*p* < 0.001). However, the treatment groups that received the ethanol-extracted *V. uliginosum* samples (EL, EM, EH) showed a decrease in skin thickness compared to the CON group (Figure 3C: *p* < 0.001). According to the analysis of collagen contents by IHC staining, compared with the abundance, and density of collagen fibers in the dermis of the NOR group, it was found to be significantly reduced in both the CON group (Figure 3D: *p* < 0.05). However, administration with high dose of ethanol-extracted *V. uliginosum* significantly inhibited the UVB irradiation-induced loss of collagen fibers compared to the CON group (Figure 3D: *p* < 0.05).

### 3.5. Effects of an Anthocyanin-Enriched Extract from V. uliginosum on mRNA Expression

**Figure 4:**
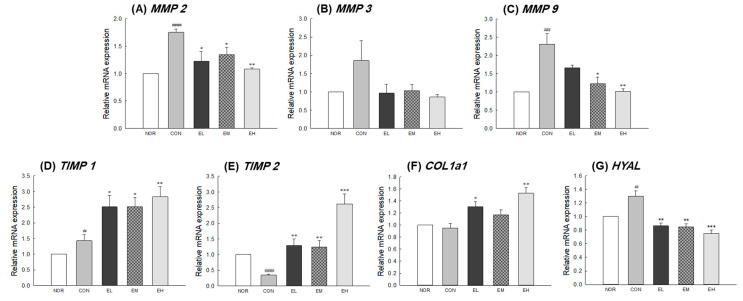
Effects of an anthocyanin-enriched extract from *Vaccinium uliginosum* on mRNA expression of (**A**–**C**) matrix metalloproteinases (*MMP*s), (**D**,**E**) tissue inhibitor of metalloproteinases (*TIMP*s), (**F**) collagen type I alpha 1 chain (*COL1a1*) and (**G**) hyaluronidase (*HYAL*) in UVB-irradiated mouse skin. NOR: normal group; CON: UVB-control group; EL: low dose of ethanol-extracted *V. uliginosum-*treated group; EM: middle dose of ethanol-extracted *V. uliginosum-*treated group; EH: high dose of ethanol-extracted *V. uliginosum-*treated group. Values are means ± standard error of the mean (SEM) for each group. Symbols indicate significant differences at ^#^ *p* < 0.05, ^##^ *p* < 0.01, and ^###^ *p* < 0.001 vs. NOR group and * *p* < 0.05, ** *p* < 0.01, and *** *p* < 0.001 vs. CON group.

**Figure 5:**
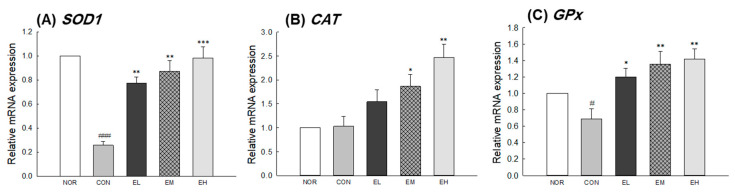
Effects of an anthocyanin-enriched extract from *Vaccinium uliginosum* on antioxidant-related mRNA expression of (**A**) *SOD1*, (**B**) *CAT,* and (**C**) *GPx* in UVB-irradiated mouse skin. NOR: normal group; CON: UVB-control group; EL: low dose of ethanol-extracted *V. uliginosum-*treated group; EM: middle dose of ethanol-extracted *V. uliginosum-*treated group; EH: high dose of ethanol-extracted *V. uliginosum-*treated group. Values are means ± standard error of the mean (SEM) for each group. Symbols indicate significant differences at ^#^ *p* < 0.05 and ^###^ *p* < 0.001 vs. NOR group and * *p* < 0.05, ** *p* < 0.01, and *** *p* < 0.001 vs. CON group.

To investigate the effect of *V. uliginosum* on skin condition-related genes, mRNA expression of *MMPs, TIMPs*, *COL1a1,* and *HYAL* in mouse skin tissue was measured by qRT-PCR analysis. There were significant differences between the NOR and CON groups (Figure 4A,E: *p* < 0.001; Figure 4C: *p* < 0.01; Figure 4D,G: *p* < 0.05) except for the expression of *MMP-3* and *COL1a1* (Figure 4B,F). The EL, EM, and EH groups showed decreased *MMP-2* expression in the skin compared to the CON group (EL and EM: *p* < 0.05; EH: *p* < 0.01). The *MMP-9* expression significantly increased in CON group compared with NOR group and decreased in the EM and EH groups compared with CON group (EM: *p* < 0.05; EH: *p* < 0.01). Relative mRNA expression level of *TIMP-1* and *TIMP-2* were significantly higher in the skin from the EL, EM, and EH groups than in that from the CON group (Figure 4D: *p* < 0.05 and *p* < 0.01; Figure 4E: *p* < 0.01 and *p* < 0.001). In addition, it was confirmed that the expression of collagen type I alpha 1 chain (*COL1a1*) related to elasticity was not regulated by UVB irradiation but was significantly increased by an anthocyanin-enriched extract (Figure 4F, EL: *p* < 0.05; EH: *p* < 0.01). In the case of hyaluronidase, which catalyzes the degradation of hyaluronic acid, it was significantly elevated in the CON group receiving only UVB irradiation, but significantly decreased in the group receiving an anthocyanin-enriched extract (Figure 4G, EL and EM: *p* < 0.01; EH: *p* < 0.001). In addition, in the case of expression of antioxidant-related genes, SOD 1 and GPx significantly decrease in CON group compared with NOR group (*p* < 0.001 and *p* < 0.05) and increased in the groups treated an anthocyanin-enriched extract compared with CON group (Figure 5A: *p* < 0.01 and *p* < 0.001; Figure 5C: *p* < 0.05 and *p* < 0.01). *CAT* gene expression was not significantly different in the NOR group and the CON group, but showed a significant increase in the EM and EH groups when compared to the CON group (Figure 5B: *p* < 0.05 and *p* < 0.01).

### 3.6. Effects of an Anthocyanin-Enriched Extract from V. uliginosum on MAPK Phosphorylation

**Figure 6:**
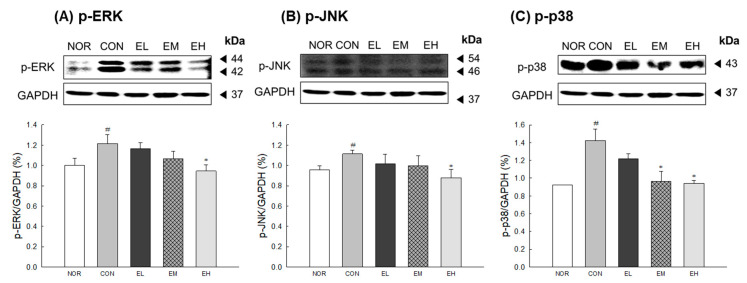
Effects of an anthocyanin-enriched extract from *Vaccinium uliginosum* on the protein level of (**A**) c-Jun N-terminal kinase (p-JNK), (**B**) extracellular signal regulated kinase (p-ERK), and (**C**) p-p38 in UVB-irradiated mouse skin. Western blot and protein quantifications are shown. NOR: normal group; CON: UVB-control group; EL: low dose of ethanol-extracted *V. uliginosum-*treated group; EM: middle dose of ethanol-extracted *V. uliginosum-*treated group; EH: high dose of ethanol-extracted *V. uliginosum-*treated group. Values are means ± standard error of the mean (SEM) for each group. Symbols indicate significant differences at ^#^ *p* < 0.05 vs. NOR group and * *p* < 0.05 vs. CON group.

To assess the effects of *V. uliginosum* on the protein level of MAPKs, Western blot analysis was performed. The protein levels of p-JNK and p-ERK in the CON group were significantly increased than the NOR group (Figure 6A,B: *p* < 0.05). The ethanol-extracted *V. uliginosum* sample (EH) showed a decrease in the protein levels of p-JNK and p-ERK compared to the CON group (*p* < 0.05). The CON group had a significantly increased expression of p-p38 compared to the NOR group (Figure 6C: *p* < 0.05); expression of p-p38 in *V. uliginosum*-treated groups, aside from the EL and EH groups, were significantly decreased compared with CON group (*p* < 0.05).

### 3.7. Effects of an Anthocyanin-Enriched Extract from V. uliginosum on Cytokine Levels

**Figure 7:**
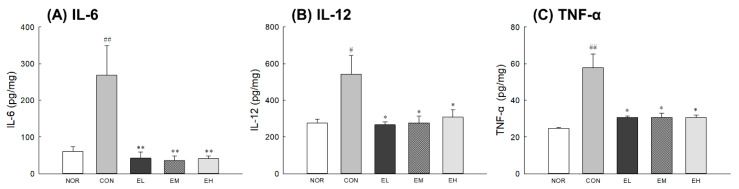
Effects of an anthocyanin-enriched extract from *Vaccinium uliginosum* on cytokine level of (**A**) IL-6, (**B**) IL-12, and (**C**) TNF-α in UVB-irradiated mouse skin. NOR: normal group; CON: UVB-control group; EL: low dose of ethanol-extracted *V. uliginosum-*treated group; EM: middle dose of ethanol-extracted *V. uliginosum-*treated group; EH: high dose of ethanol-extracted *V. uliginosum-*treated group. Values are means ± standard error of the mean (SEM) for each group. Symbols indicate significant differences at ^#^ *p* < 0.05 and ^##^ *p* < 0.01 vs. NOR group and * *p* < 0.05 and ** *p* < 0.01 vs. CON group.

Inflammatory cytokine levels were significantly increased in CON group compared with NOR group (Figure 7A,C: *p* < 0.001; Figure 7B: *p* < 0.05). The administration of an anthocyanin-enriched extract from *V. uliginosum* was related to a decrease in cytokine levels of UV-damaged skin tissues. The groups administrated orally with an anthocyanin-enriched extract from *V. uliginosum* had significantly lower IL-6 (Figure 7A: *p* < 0.01), IL-12 (Figure 7B: *p* < 0.05), and TNF- α (Figure 7C: *p* < 0.05) levels compared to the CON group. Although UV irradiation increased the levels of various inflammatory mediators, administration of an anthocyanin-enriched extract inhibited the increase in UV-induced cytokine levels.

## 4. Discussion

UVB irradiated skin is characterized by wrinkles, roughness, fine lines, increased coarseness, and irregular pigmentation [16]. UVB irradiation affects various skin cell functions through DNA damage and ROS generation in the skin. The excessive production of ROS is involved in protein oxidation and lipid peroxidation, which accelerate skin aging by decomposing collagen and elastin fibers, matrix components of the dermal layer, by promoting the expression of MMPs [17]. Many natural products have demonstrated protective and antiaging effects on the skin through ROS scavenging.

It has been shown to improve protection against skin aging through dietary supplementation containing vitamins C and E, carotenoids, coenzyme Q10, collagen peptides, and polyunsaturated fatty acids [18,19]. Plant extracts containing large amounts of phytochemicals such as flavonoids and carotenoids are also consumed for this purpose [20].

Here, we evaluated the effects of *V. uliginosum* on skin damaged by UVB. UVB irradiation leads to an increase in skin dehydration, TEWL, erythema, skinfold thickness, and epidermal thickness (Figure 1), all of which are indicators of skin barrier function [21,22]. *V. uliginosum* treatment effectively reduced skin damage by increasing the change in skin hydration and decreasing the change in TEWL, erythema value, skinfold thickness, and epidermal thickness following UVB irradiation. Antioxidants have known effects on epidermal barrier function in both healthy and compromised skin [23]. Nutrients such as anthocyanins contained *V. uliginosum* extract seem to have an effect on the skin defenses against oxidative stress.

We also analyzed a replica of mouse dorsal skin tissue to evaluate the effects of an anthocyanin-enriched extract from *V. uliginosum* on wrinkle formation (Figure 2). UVB irradiation makes the skin epidermis rough and decreases normal collagen and elastin structures, resulting in loss of elasticity due to a decrease in ECM proteins [24]. UVB-generated ROS results in increased transcription of the MMP encoding genes [25]. MMP expression induced by UVB exposure contributes to the skin wrinkle formation by promoting the degradation of ECM, including collagen fibers [26]. An anthocyanin-rich extract has phytoestrogenic activities and is known to upregulate the expression of collagen and elastin [10]. In addition, various studies to address the above problems have shown that dietary supplements containing vitamins, antioxidants, collagen peptides, and pre- and probiotics have positive effects on skin elasticity [27,28,29].

We investigated the photoprotective effects of *V. uliginosum* extract on mRNA expression of *MMPs, TIMPs*, *COL1a1*, *HYAL*, *SOD1*, *CAT,* and *GPx* in UVB-damaged skin samples (Figure 4 and Figure 5). *V. uliginosum* attenuated the UVB-induced *MMP* expression and increased *TIMP*, *COL1a1*, *HYAL*, *SOD1*, *CAT,* and *GPx* expression. Intracellular ROS regulate the transcription of MMPs through activation of MAPK and NF-κB, ultimately leading to oxidative damage of DNA, proteins, and lipids [30]. Typical defense mechanisms against increased ROS in the skin include enzymes such as SODs, CAT, GPx, and peroxiredoxins, and nonenzymatic antioxidants [31]. The antioxidant enzyme systems, increased through botanical extracts or natural ingredients, are known to reduce UV-mediated ROS production and regulate MMPs and MAPK signaling. The anthocyanins from purple-fleshed sweet potato and *V. uliginosum* attenuated UV-induced toxicity involving ROS formation, lipid peroxidation, and inflammation in BALB/c-nu mice skin and human dermal fibroblasts [32,33]. Members of the flavonoid group of phytochemicals have been reported to reduce ROS produced by stress in human dermal fibroblasts and animal models and to regulate the expression of genes related to wrinkle development and morphology [8,34]. In addition, botanical extracts have been shown to prevent or alleviate skin wrinkles caused by the secretion of inflammatory cytokines while promoting collagen production. Therefore, based on our results, we hypothesize that *V. uliginosum* extract effectively prevents skin photoaging by reducing the production of MMPs [35,36,37].

We also investigated effects of *V. uliginosum* administration on the UVB-induced phosphorylation of MAPKs and the production of cytokines (Figure 6 and Figure 7). Most skin-damaging external stimuli, including UV irradiation, are involved in the degradation of the ECM by regulating the MAPK pathway, including the proteins ERK, JNK, and p38 kinase, and by regulating the expression and activity of Jun and Fos [38]. MAPKs are known to regulate the expression of MMPs; the MEK/ERK signaling pathway is directly related to regulate MMP production in human dermal fibroblasts [39]. In addition, increased inflammatory cytokines stimulate keratinocytes and dermal fibroblasts to produce MMPs and induce the degradation of ECM proteins [40]. An anthocyanin-enriched extract from *V. uliginosum* significantly decreased inflammatory cytokine levels and reduced phosphorylation due to UV radiation-induced oxidative stress of ERK, JNK, and p38 proteins. Similar to our results, anthocyanins from black peanut and rose petal extracts showed skin-protective effect and anti-inflammatory activity through activation of antioxidant response factor-related genes and reduction of phospho-MAPK levels [41,42]. These results suggest that ethanol-extracted *V. uliginosum* extract has an inhibitory effect on UV radiation-induced MMP-1 production and oxidative stress by regulating changes in the MAPK signaling pathway.

## 5. Conclusions

Botanical extracts could contain various active compounds that provide antioxidant capacities and health enhancement of possibilities. The fruit of *V. uliginosum,* with a high level of anthocyanins, is also high in antioxidants and has potential for use in dietary supplement for skin health. In this study, the extracts from *V. uliginosum* containing a high level of anthocyanins showed a photoprotective effect against UVB-induced skin damage in a hairless mouse model by inhibition of MMP expression and pro-inflammatory cytokines through increased antioxidant enzyme gene expression and reduced phospho-MAPK levels.

## Figures and Tables

**Table 1:** Anthocyanin content and radical scavenging activity of ethanol-extracted *V. uliginosum* samples.

Process	Polyphenol (μg/mg)	Cyanidin-3-*O*-Glucoside (μg/g)	Delphinidin-3-*O*-Glucoside (μg/g)	Malvidin-3-*O*-Galactoside (μg/g)	ABTS IC_50_ (mg/mL)	DPPH IC_50_ (mg/mL)	FRAP (mM)
After extraction	44.37 ± 0.30	127.9 ± 16.10	572.14 ± 73.03	58.62 ± 7.81	1.93 ± 0.11	2.44 ± 0.09	0.20 ± 0.00

Values are means ± standard deviation.

## Supplementary Materials

Supplementary Materials can be found at https://www.mdpi.com/2076-3921/9/9/844/s1. Table S1. Food intake, drinking volume and body weight. Figure S1. Effects of an anthocyanin-enriched extract from *Vaccinium uliginosum* on UVB-irradiated mouse skin.
