# Supplementary material for: An Anthocyanin-Enriched Extract from Vaccinium uliginosum Improves Signs of Skin Aging in UVB-Induced Photodamage
Source: Antioxidants (Basel). 2020 Sep 9;9(9):844. doi: 10.3390/antiox9090844 (PMC7554747; doi:10.3390/antiox9090844)
Supplement: Supplementary file 1 [file antioxidants-09-00844-s001.pdf]

Table S1. Food intake, drinking volume and body weight.

Group	NOR	CON	EL	EM	EH
Food intake (g/day)	5.00 ± 0.10 ^{ns}	6.79 ± 0.48	5.21 ± 0.05	4.96 ± 0.16	5.45 ± 0.11
Drink volume (mL/day)	4.29 ± 0.11 ^{ns}	4.86 ± 0.11	5.33 ± 0.22	5.29 ± 0.03	4.86 ± 0.16
Average daily gain (mg/day)	125.01 ± 7.17 ^{ns}	122.92 ± 12.33	122.08 ± 10.43	121.83 ± 14.48	123.75 ± 11.07

NOR: normal group; CON: UVB-control group; EL: low dose of ethanol-extracted *V. uliginosum*-treated group; EM: middle dose of ethanol-extracted *V. uliginosum*-treated group; EH: high dose of ethanol-extracted *V. uliginosum*-treated group. Data are expressed as mean ± standard error (n=6), NS: not significant.

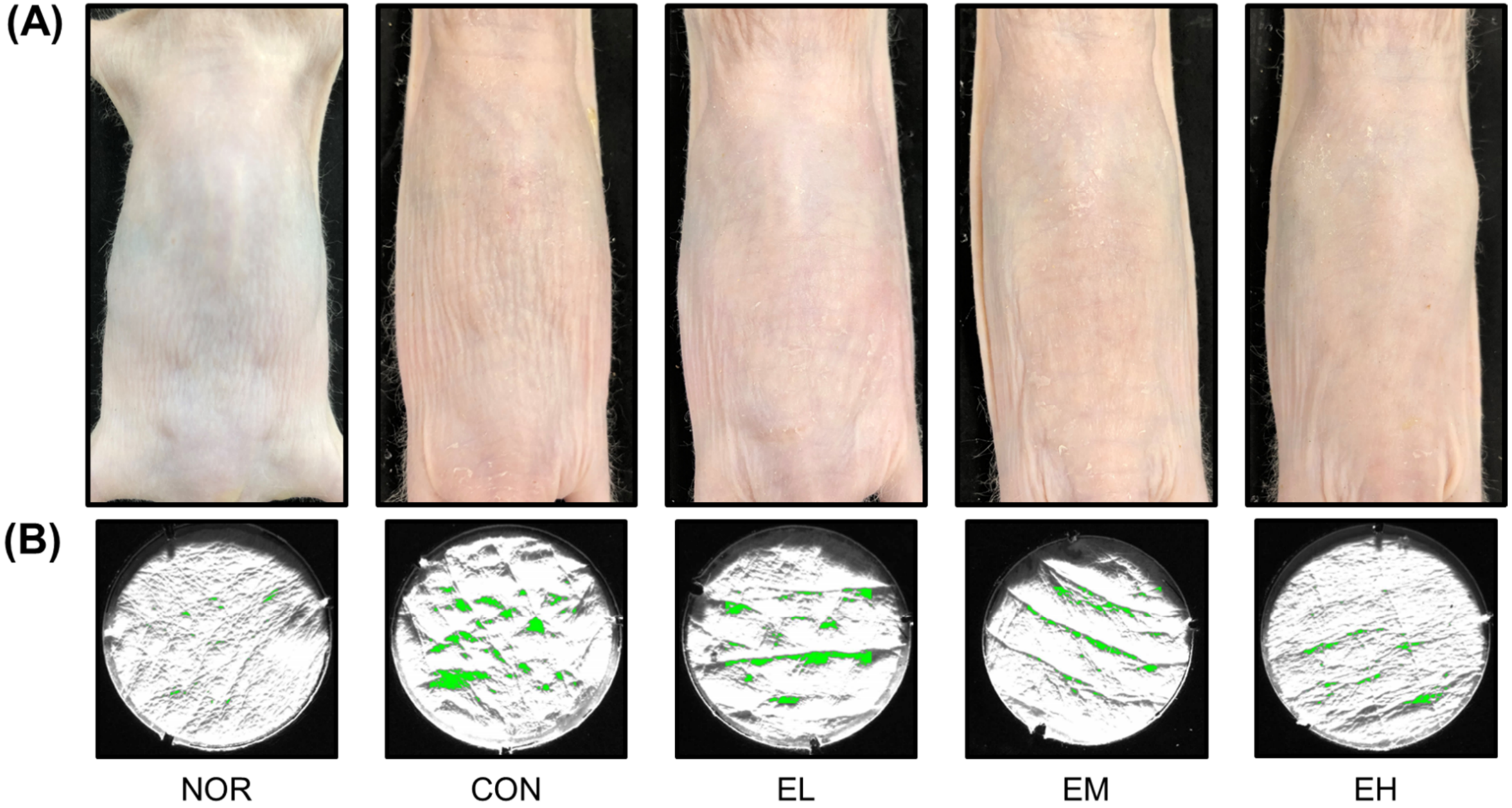

Figure S1. Effects of an anthocyanin-enriched extract from *Vaccinium uliginosum* on UVB-irradiated mouse skin. The dorsal skin of hairless mice was (A) photographed, and (B) replicas of the back skin were made using a Replica full kit. NOR: n ormal group; CON: UVB-control group; EL: low dose of ethanol-extracted *V. uliginosum*-treated group; EM: middle dose of ethanol-extracted *V. uliginosum*-treated group; EH: high dose of ethanol-extracted *V. uliginosum*-treated group.
